# Concordance of Cancer Screening Attendance Among Spouse Couples: A Cross‐Sectional Survey of the Tohoku Medical Megabank Project

**DOI:** 10.1002/pon.70158

**Published:** 2025-04-25

**Authors:** Naoki Nakaya, Kumi Nakaya, Toshimasa Sone, Mana Kogure, Rieko Hatanaka, Ippei Chiba, Sayuri Tokioka, Masato Takase, Yoko Izumi, Nobuo Fuse, Atsushi Hozawa

**Affiliations:** ^1^ Tohoku Medical Megabank Organization Tohoku University Sendai Japan; ^2^ Tohoku University Graduate School of Medicine Sendai Japan; ^3^ Department of Occupational Therapy School of Health Sciences Fukushima Medical University Fukushima Japan

**Keywords:** cancer, cancer screening, concordance, cross‐sectional study, odds ratio, oncology, spousal pairs

## Abstract

**Objectives:**

Owing to spousal pairs often exhibiting similar health behaviors, this study examined the concordance of cancer screening attendance between spouses using cross‐sectional data from a large biobank study in Japan, which included 2022 spousal pairs.

**Study Design:**

Cross‐sectional study.

**Methods:**

Self‐administered data were collected to determine whether participants had undergone screening for colorectal, gastric, and lung cancers in the past year. The following two analyses were conducted: the exposure was whether the husband attended cancer screening, and the outcome was whether the wife attended; the exposure was whether the wife attended, and the outcome was whether the husband attended. Multiple logistic regression analyses were performed, adjusting for confounding factors in the exposed individuals.

**Results:**

The multivariate odds ratio (95% confidence interval, *p*‐value) for wives attending colorectal cancer screening when their husbands had attended was 2.7 (2.2–3.3, *p* < 0.0001), indicating a significant positive association. Similarly, when wives were the exposure and husbands were the outcomes, the odds ratio was 2.6 (2.2–3.2, *p* < 0.0001). Notably, these associations were consistent across colorectal, gastric, and lung cancer screenings.

**Conclusions:**

The findings of this study support the hypothesis that the attendance of one spouse at cancer screening significantly positively influences that of the other spouse, regardless of the type of cancer screening or the age of the spouses. Novel intervention strategies can be developed that specifically target spousal pairs and potentially enhance the effectiveness of cancer prevention initiatives compared to those targeting individuals alone.

## 1 Introduction

The health and diseases of one spouse are closely linked to those of the other, highlighting the importance of spousal health in epidemiological studies. These studies have typically been based on two main hypotheses. The first hypothesis suggests that a serious health outcome in one spouse, such as illness or death, negatively impacts the health of the other spouse [1, 2, 3, 4, 5], possibly due to stress mechanisms such as psychosocial [6] and caregiving burdens [7]. The second hypothesis posits that spouses tend to have similar lifestyle habits, health status, disease prevalence, and disease onset [8, 9, 10, 11, 12], potentially due to cohabitation effects or assortative mating, where couples share similar hobbies and characteristics [13]. In line with the second hypothesis, spouses may also exhibit similar health‐seeking behaviors, including attendance at primary (e.g., health checkup attendance) and secondary prevention activities (e.g., cancer screening attendance).

Cancer screening facilitates early detection and treatment, which reduces mortality rates. In Japan, the Health Promotion Act promotes five types of cancer screenings: colorectal, stomach, lung, breast, and cervical cancers [14]. However, screening attendance remains low. The attendance rate for all five cancers is below 60% [15, 16], which is lower than that in other countries, necessitating strategies to increase participation.

Similarities exist in cancer screening attendance among spouses. For example, Kotwal et al. examined colorectal cancer screening (colonoscopy) concordance in a U.S. national sample of 804 spousal pairs [17], finding that if one spouse had undergone colorectal cancer screening in the past 5 years, the odds of the other spouse doing the same were 1.9 times higher compared with that of couples where one spouse had not attended the screening. Another Japanese study investigated the association between wives' attendance at cervical or breast cancer screenings and husbands' participation in health checkups. This study [18] found a significant positive association and involved approximately 40,000 couples.

Despite these findings, further studies must explore spousal concordance in health behaviors. Specifically, a larger sample size is required to examine the association between spousal cancer screening attendance, especially for screenings beyond colorectal cancer.

This study aimed to analyze the concordance of spousal pairs for colorectal, stomach, and lung cancer screening using a cross‐sectional dataset from a community population in Japan. The study included approximately 2000 couples, and the analysis accounted for strict confounding factors. The study established a clear association, which may provide insights for developing future intervention strategies, specifically for spousal pairs, potentially enhancing the effectiveness of cancer prevention initiatives beyond those targeting individuals alone.

## 2 Methods

### 2.1 Study Setting

For this cross‐sectional study, data were obtained from the Tohoku Medical Megabank (TMM) Community‐based Cohort Study (TMM CommCohort Study) conducted in Miyagi Prefecture, northern Japan [8, 9, 19, 20, 21]. The baseline survey for the TMM CommCohort Study was conducted between May 2013 and March 2016 and employed two recruitment approaches. First, participants aged 40–74 years were recruited from the annual community health examinations run by local governments for insured persons in Miyagi Prefecture (Type 1 survey). Second, seven Community Support Center facilities in Miyagi Prefecture recruited and assessed residents aged ≥ 20 years through voluntary admission (Type 2 survey). During the baseline survey, blood and urine samples were collected, and self‐administered questionnaires covering lifestyle habits, medical histories, and family relationships were completed by participants in the Type 1 and 2 surveys. Additional physiological examinations and a self‐response questionnaire using a touchscreen PC were conducted exclusively in Type 2 surveys. Some Type 1 participants later visited the Community Support Center facilities for more detailed examinations and completed the touchscreen questionnaire. This study exclusively utilized data from the Type 2 survey. Written informed consent was obtained from participants.

Self‐administered family relationship questionnaires were distributed and collected. Participants were asked, “If you are living with family members who are participating in this TMM project, please specify all their names and birthdays and your relationships with them (your spouse, father, mother, children, grandchildren, children's spouses, father‐in‐law, mother‐in‐law, and others) with their consent.” A participant and their spouse were defined as a spousal pair if the spouse listed in the family relationship was identified in the TMM CommCohort Study [8, 9].

### 2.2 Self‐Administered Cancer Screening Attendance

Using a touchscreen PC, participants were asked: “In the past year, did you attend a medical checkup or cancer screening at your local government office, your workplace, a hospital, or another facility?” Multiple responses were allowed, including options for gastric, lung, colorectal, breast, prostate, and cervical cancer screenings, along with general and specific health checkup questions. Regarding specific health checkup, all health insurers in Japan provide health checkup programs to all enrollees and their dependents aged 40–74 years, along with lifestyle improvement counseling for nonmedicated participants who have elevated risk factors of metabolic syndrome [22]. This study focused on gastric, colorectal, and lung cancer screenings, specifically for spouses who attended these screenings within the past year (Supporting Information S1). In Japan, cancer screenings are categorized into “municipality‐based,” “workplace‐based,” and “voluntary” screenings.

### 2.3 Possible Confounding Factors

In the analyses of concordance in cancer screening attendance among married couples, several potentially important confounding factors were considered and included as covariates in multivariate models. These factors were as follows:

1. Fiscal years of baseline survey (2013, 2014, or 2015).
2. Educational level (low: elementary school, junior high school, or high school; medium: vocational school, college, or technical college; high: university or graduate school; unknown).
3. Working status (regular employee, non‐regular employee, not working, or unknown).
4. Number of cohabitants (fewer than two people, three or more people, or unknown).
5. Smoking status (never smokers, current smokers, past smokers, or unknown).
6. Alcohol consumption (never drinkers, current drinkers, past drinkers, or unknown).
7. Twenty‐four‐hour urinary sodium excretion [23] (less than median, median, more than median, or unknown) (Supporting Information S1).
8. Physical activity (PA) in metabolic equivalents (METs) hours/day [8, 9] (less than median, median, more than median, or unknown) (Supporting Information S1).
9. Body mass index (BMI) in kg/m^2^ (underweight: < 18.5, normal weight: 18.5–24.9, overweight: ≥ 25.0 or unknown).
10. Comorbidities (self‐reported cancer, heart disease, or stroke).
11. Depressive symptoms (none: Center for Epidemiological Studies Depression Scale [CES‐D] score < 16, present: CES‐D score > 16, or unknown) [24, 25].
12. Social network (Lubben Social Network Scale Item 2[26, 27]: fewer than two people, three or more people, or unknown).

The age of each participant at the baseline survey and fiscal year was obtained from the consent forms. Data on educational level, working status, number of cohabitants, smoking status, alcohol consumption, PA, comorbidities, depressive symptoms, and social networks were collected through self‐administered questionnaires. Information regarding 24‐h urinary sodium excretion was obtained from consent forms, urine tests, and physiological tests (Supporting Information S1). BMI was calculated using data obtained from physiological tests.

### 2.4 Statistical Analyses

First, the mean (standard deviation) and percentage of potential confounding factors for each spouse (husband or wife) were calculated based on whether they had undergone screening for colorectal, gastric, and lung cancers. Next, a 2 × 2 contingency table was created to determine whether both spouses had attended cancer screenings. Herein, the following two analyses were performed: one where the exposure was whether the husband had attended cancer screening, and the outcome was whether the wife had attended, and the other where the roles were reversed. For each analysis, the following three models were developed: (a) an unadjusted (crude) model, (b) a model adjusted for the husband's age at baseline survey as the exposure, and (c) a model adjusted for confounding factors specific to the husband's exposure. The analyses were repeated with the wife as the exposure and the husband as the outcome using the same models ([a] to [c]). Third, stratified analyses were performed based on age at the time of the survey, and the participants were divided into two groups: those aged 64 years or younger and those aged 65 years or older. Given the high correlation coefficient for the couple's ages (0.92), the analysis was stratified by the age of one spouse only.

Logistic regression analyses were used to calculate spousal concordance for the categorical variables. The odds ratio (OR) (95% confidence interval [CI]) was calculated for one spouse attending cancer screening based on the screening status of the other spouse. All statistical analyses were conducted using SAS software (version 9.4; SAS Institute Inc., Cary, NC, USA), with statistical significance set at *p* < 0.05.

## 3 Results

**FIGURE 1:**
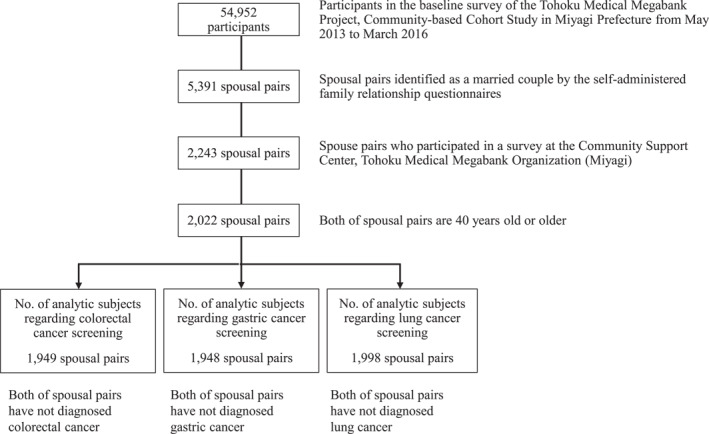
Flowchart of inclusion of participants and analysis in this study.

This study included 54,952 participants from the baseline survey of the TMM CommCohort Study in Miyagi Prefecture. Among these participants, 5391 spousal pairs were identified through the self‐administered family relationship questionnaire, and 2243 of these pairs underwent detailed examinations at Community Support Center facilities during the baseline survey. Since colorectal, gastric, and lung cancer screenings are recommended in Japan for people aged ≥ 40 years, the analysis was limited to spousal pairs within this age group, totaling 2022 pairs. Further screening‐specific analyses were restricted to spouses who had not been affected by the respective cancers: 1949 for colorectal cancer, 1948 for gastric cancer, and 1998 for lung cancer (Figure 1).

The potential confounding factors regarding attendance at colorectal, gastric, and lung cancer screenings were assessed for each spouse. For both husbands and wives, those who attended screenings tended to be older, more likely to be non‐regular employees or not working, less likely to be current smokers, more physically active, and more likely to have a BMI within the appropriate range of 18.5–25.0, compared with those who did not attend screenings (Table 1).

**TABLE 1:** Demographic characteristics of participants who underwent cancer screening with spousal pairs according to cancer screening attendance.

Variables	Colorectal cancer screening (1949 spousal pairs)								Gastric cancer screening (1948 spousal pairs)								Lung cancer screening (1998 spousal pairs)							
	Husbands				Wives				Husbands				Wives				Husbands				Wives			
	Attended (*n* = 889)		Non‐attended (*n* = 1060)		Attended (*n* = 1020)		Non‐attended (*n* = 929)		Attended (*n* = 977)		Non‐attended (*n* = 971)		Attended (*n* = 959)		Non‐attended (*n* = 989)		Attended (*n* = 745)		Non‐attended (*n* = 1253)		Attended (*n* = 712)		Non‐attended (*n* = 1286)	
Mean age (±S.D.) at baseline survey (years)																								
	66.1	±7.7	63.1	±9.6	65.3	±8.2	63.5	±9.5	66.1	±7.9	62.7	±9.5	65.6	±8.3	63.3	±9.3	66.9	±7.2	63.1	±9.4	64.8	±8.3	64.4	±9.2
Fiscal years of baseline survey (%)																								
2013	299	34	275	26	335	33	266	29	333	34	240	25	333	35	267	27	244	33	344	27	216	30	401	31
2014	319	36	380	36	362	35	336	36	335	34	360	37	332	34	370	37	264	35	449	36	244	34	464	36
2015	271	30	405	38	323	32	327	35	309	32	371	38	304	32	352	36	237	32	460	37	252	35	421	33
Educational level (%)																								
Low level	544	61	585	55	614	60	567	61	584	60	545	56	566	59	603	61	449	60	710	57	417	59	795	62
Medium level	78	9	123	12	328	32	278	30	79	8	119	12	307	32	307	31	61	8	141	11	232	33	388	30
High level	254	29	340	32	69	7	79	9	299	31	291	30	78	8	72	7	220	30	385	31	56	8	95	7
Unknown	13	1	19	2	9	1	5	1	15	2	16	2	8	1	7	1	15	2	17	1	7	1	8	1
Working status (%)																								
Regular employee	135	15	289	27	54	5	60	6	155	16	273	28	51	5	65	7	92	12	339	27	29	4	90	7
Non‐regular employee or not working	735	83	759	72	956	94	858	92	806	83	686	71	899	94	912	92	637	86	899	72	678	95	1179	92
Unknown	19	2	12	1	10	1	11	1	16	2	12	1	9	1	12	1	16	2	15	1	5	1	17	1
Cohabitants (%)																								
≤ 2	480	54	486	46	530	52	436	47	516	53	448	46	512	53	452	46	413	55	579	46	376	53	619	48
≥ 3	392	44	549	52	484	47	477	51	439	45	502	52	440	46	521	53	314	42	648	52	330	46	649	50
Unknown	17	2	25	2	6	1	16	2	22	2	21	2	7	1	16	2	18	2	26	2	6	1	18	1
Smoking status (%)																								
Never smokers	243	27	273	26	920	90	769	83	276	28	243	25	869	91	818	83	196	26	338	27	620	87	1113	87
Current smokers	113	13	211	20	31	3	42	5	125	13	200	21	26	3	46	5	93	12	236	19	29	4	44	3
Past smokers	530	60	572	54	65	6	113	12	571	58	525	54	62	6	118	12	455	61	672	54	61	9	121	9
Unknown	3	0	4	0	4	0	5	1	5	1	3	0	2	0	7	1	1	0	7	1	2	0	8	1
Alcohol drinking status (%)																								
Never drinkers	138	16	196	18	578	57	522	56	151	15	179	18	541	56	556	56	123	17	220	18	372	52	757	59
Current drinkers	712	80	822	78	430	42	396	43	790	81	752	77	410	43	419	42	595	80	981	78	334	47	511	40
Past drinkers	39	4	41	4	11	1	11	1	36	4	39	4	7	1	14	1	27	4	51	4	5	1	18	1
Unknown	0	0	1	0	1	0	0	0	0	0	1	0	1	0	0	0	0	0	1	0	1	0	0	0
24‐h urinary sodium excretion in grams/day (%)																								
< Median	427	48	543	51	492	48	479	52	471	49	498	51	469	49	502	51	372	50	621	50	347	49	648	50
≥ Median	460	52	507	48	527	52	443	48	503	51	466	48	488	51	482	49	370	50	623	50	363	51	632	49
Unknown	2	0	10	1	1	0	7	1	3	0	7	1	2	0	5	1	3	0	9	1	2	0	6	3
Physical activity in METs hours/day (%)																								
< Median	391	44	564	53	484	47	445	48	429	44	524	54	460	48	473	48	327	44	651	52	337	47	618	48
≥ Median	472	53	477	45	505	50	455	49	524	54	427	44	473	49	483	49	403	54	574	46	356	50	626	49
Unknown	26	3	19	2	31	3	29	3	24	2	20	2	26	2	33	3	15	2	28	2	19	3	42	3
Body mass index in kg/m^2^ (%)																								
< 18.5	20	2	23	2	87	9	89	10	17	2	23	2	76	8	99	10	18	2	28	2	61	9	122	9
18.5–24.9	599	67	678	64	717	70	633	68	661	68	610	63	685	71	671	68	498	67	804	64	508	71	879	68
≥ 25.0	260	29	354	33	212	21	191	21	290	30	325	33	194	20	204	21	224	30	402	32	140	20	268	21
Unknown	10	1	14	1	4	0	16	2	9	1	13	1	4	0	15	2	5	1	19	2	3	0	17	1
Past histories of chronic diseases (%)																								
Cancer	79	9	71	7	82	8	61	7	61	6	43	4	53	6	51	5	62	8	85	7	49	7	98	8
Stroke	42	5	42	4	11	1	13	1	44	5	39	4	13	1	13	1	36	5	52	4	4	1	24	2
MI or angina pectoris	50	6	63	6	20	2	12	1	54	6	59	6	17	2	17	2	42	6	77	6	15	2	20	2
Depressive symptoms (CES‐D) (%)																								
Score < 16	744	84	891	84	804	79	683	74	830	85	806	83	764	79	729	74	638	86	1040	83	571	80	971	76
Score ≥ 16	126	14	149	14	192	19	213	23	127	13	148	15	183	19	231	23	95	13	186	15	133	19	282	22
Unknown	19	2	20	2	19	2	27	3	20	2	17	2	12	2	29	3	12	2	27	2	8	1	33	3
Item 2 of the Lubben Social Network Scale: How many relatives do you feel close to, such that you can talk about private matters? (%)																								
≤ 2	82	9	113	11	77	8	85	9	98	10	100	10	77	8	87	9	74	10	134	11	51	7	116	9
≥ 3	796	90	927	87	929	91	832	90	864	88	856	88	867	90	891	90	659	88	1100	88	654	92	1150	89
Unknown	26	3	19	2	31	3	29	3	24	2	20	2	26	2	33	3	15	2	28	2	19	3	42	3

*Note:* Median 24‐h urinary sodium excretion in grams/day: 9.74 in men, 10.38 in women for colorectal cancer screening, 9.73 in men, 10.38 in women for gastric cancer screening, 9.75 in men, 10.38 in women for lung cancer screening. Median physical activity in METs hours/day: 16.51 in men, 13.95 in women for colorectal cancer screening, 16.49 in men, 13.95 in women for gastric cancer screening, 16.54 in men, 13.95 in women for lung cancer screening.

Abbreviations: CES‐D, Center for Epidemiological Studies Depression Scale; METs, metabolic equivalents; MI, myocardial infarction; S.D., standard deviation.

For colorectal cancer screening, the crude OR of wives attending screening when their husbands attended was 2.8 (95% CI: 2.4–3.4, *p* < 0.0001). This association remained statistically significant after adjusting for the husband's age at the baseline survey (OR: 2.7, 95% CI: 2.3–3.3, *p* < 0.0001) and after further adjusting for additional confounders (OR: 2.7 95%: 2.2–3.3, *p* < 0.0001). Similarly, when the analysis was conducted with wives as the exposure and husbands as the outcome, the crude OR for husbands attending screening when their wives attended was also 2.8 (95% CI: 2.4–3.4, *p* < 0.0001). This association remained significant after adjusting for the wife's age (OR: 2.7, 95% CI: 2.3–3.3, *p* < 0.0001) and additional confounders (OR: 2.6, 95% CI: 2.2–3.2, *p* < 0.0001).

These patterns were consistent across screenings for gastric and lung cancers. Spouses were significantly more likely to attend screenings if their partner attended, regardless of which spouse was considered the exposure (Table 2). Table 3 presents the main results stratified by age of the exposed spouse at baseline. The results remained consistent across all types of cancer screening, regardless of whether husbands or wives were considered the exposure (Table 3).

**TABLE 2:** Associations between cancer screening attendance of one spouse and the other in a pair.

Cancer screening		Colorectal cancer screening (1949 pairs)				Gastric cancer screening (1948 pairs)				Lung cancer screening (1998 pairs)			
		Husbands: Exposure		Wives: Exposure		Husbands: Exposure		Wives: Exposure		Husbands: Exposure		Wives: Exposure	
		Wives: Outcome		Husbands: Outcome		Wives: Outcome		Husbands: Outcome		Wives: Outcome		Husbands: Outcome	
	No. of exposed spouses	No. of exposed spouses		No. of exposed spouses		No. of exposed spouses		No. of exposed spouses		No. of exposed spouses		No. of exposed spouses	
	Attended	Non‐attended	Attended	Non‐attended	Attended	Non‐attended	Attended	Non‐attended	Attended	Non‐attended	Attended	Non‐attended	Non‐attended
No. of outcome spouses	Attended	588	432	588	301	628	331	628	349	347	365	347	398
	Non‐attended	301	628	432	628	349	640	331	640	398	888	365	888

	ORs (95% CIs)	*p*‐value	ORs (95% CIs)	*p*‐value	ORs (95% CIs)	*p*‐value	ORs (95% CIs)	*p*‐value	ORs (95% CIs)	*p*‐value	ORs (95% CIs)	*p*‐value
Crude model	2.8 (2.4–3.4)	< 0.0001	2.8 (2.4–3.4)	< 0.0001	3.5 (2.9–4.2)	< 0.0001	3.5 (2.9–4.2)	< 0.0001	2.1 (1.8–2.6)	< 0.0001	2.1 (1.8–2.6)	< 0.0001
Age‐adjusted model	2.7 (2.3–3.3)	< 0.0001	2.7 (2.3–3.3)	< 0.0001	3.3 (2.7–4.0)	< 0.0001	3.3 (2.7–4.0)	< 0.0001	2.1 (1.8–2.6)	< 0.0001	2.2 (1.8–2.6)	< 0.0001
Multivariate‐adjusted model	2.7 (2.2–3.3)	< 0.0001	2.6 (2.2–3.2)	< 0.0001	3.2 (2.7–3.9)	< 0.0001	3.2 (2.6–3.8)	< 0.0001	2.1 (1.7–2.6)	< 0.0001	2.2 (1.8–2.6)	< 0.0001

*Note:* The above adjustment factors were used only for spouses as exposures in the model. Median values for 24‐h urinary sodium excretion in grams/day: 9.74 in men, 10.38 in women for colorectal cancer screening, 9.73 in men, 10.38 in women for gastric cancer screening, 9.75 in men, 10.38 in women for lung cancer screening. Median values for physical activity in METs hours/day: 16.51 in men, 13.95 in women for colorectal cancer screening, 16.49 in men, 13.95 in women for gastric cancer screening, 16.54 in men, 13.95 in women for lung cancer screening. Logistic regression models were used to adjust multivariable ORs for age at baseline survey (as continuous variable), fiscal years of baseline survey (2013, 2014, or 2015), educational level (low level, medium level, high level, or unknown), working status (regular employee, non‐regular employee or not working, or unknown), cohabitants (living with fewer than two people, living with three or more people, unknown), smoking status (never smokers, current smokers, past smokers, unknown), alcohol drinking (never drinkers, current drinkers, past drinkers, unknown), 24‐h urinary sodium excretion in grams/day (less than median, median or more, or unknown), physical activity in METs hours/day (less than median, median or more, unknown), Body Mass Index in kg/m^2^ (< 18.5, 18.5–24.9, ≥ 25.0, unknown), comorbidities by self‐reported questionnaire (cancer, heart diseases, stroke disease), depressive symptoms (none [Center for Epidemiological Studies Depression Scale {CES‐D} score < 16], presence [CES‐D score ≥ 16], unknown). Item 2 of the Lubben Social Network Scale: *How many relatives do you feel close to, such that you can talk about private matters?* (fewer than two people, three or more people, and unknown).

Abbreviations: CI, confidence interval; OR, odds ratio.

**TABLE 3:** Associations between one spouse's attendance at cancer screening and the other spouse's attendance, stratified by baseline age among exposed spouses.

Cancer screening		Colorectal cancer screening (1949 pairs)				Gastric cancer screening (1948 pairs)				Lung cancer screening (1998 pairs)			
		Husbands: Exposure		Wives: Exposure		Husbands: Exposure		Wives: Exposure		Husbands: Exposure		Wives: Exposure	
		Wives: Outcome		Husbands: Outcome		Wives: Outcome		Husbands: Outcome		Wives: Outcome		Husbands: Outcome	
		No. of exposed spouses		No. of exposed spouses		No. of exposed spouses		No. of exposed spouses		No. of exposed spouses		No. of exposed spouses	
		Attended	Non‐attended	Attended	Non‐attended	Attended	Non‐attended	Attended	Non‐attended	Attended	Non‐attended	Attended	Non‐attended
Exposed spouses are 64 years or younger													
No. of outcome spouses	Attended	191	204	320	170	203	156	319	203	114	188	176	204
	Non‐attended	115	317	254	405	134	338	208	422	118	418	242	552

		ORs (95% CIs)	*p*	ORs (95% CIs)	*p*	ORs (95% CIs)	*p*	ORs (95% CIs)	*p*	ORs (95% CIs)	*p*	ORs (95% CIs)	*p*
Multivariate‐adjusted model		2.5 (1.9–3.4)	< 0.0001	2.7 (2.1–3.6)	< 0.0001	3.0 (2.3–4.1)	< 0.0001	2.8 (2.2–3.7)	< 0.0001	2.1 (1.5–2.9)	< 0.0001	2.5 (1.8–3.4)	< 0.0001
Exposed spouses are 65 years or older													
No. of outcome spouses	Attended	397	228	268	131	425	175	309	146	233	177	171	194
	Non‐attended	186	311	178	223	215	302	123	218	280	470	123	336

	ORs (95% CIs)	*p*	ORs (95% CIs)	*p*	ORs (95% CIs)	*p*	ORs (95% CIs)	*p*	ORs (95% CIs)	*p*	ORs (95% CIs)	*p*
Multivariate‐adjusted model	2.9 (2.2–3.7)	< 0.0001	2.5 (1.9–3.4)	< 0.0001	3.4 (2.6–4.4)	< 0.0001	4.0 (2.9–5.5)	< 0.0001	2.2 (1.7–2.9)	< 0.0001	1.9 (1.5–2.5)	< 0.0001

*Note:* The above adjustment factors were used only for spouses as exposures in the model. Median 24‐h urinary sodium excretion in grams/day: 9.74 in men, 10.38 in women for colorectal cancer screening, 9.73 in men, 10.38 in women for gastric cancer screening, 9.75 in men, 10.38 in women for lung cancer screening. Median physical activity in METs hours/day: 16.51 in men, 13.95 in women for colorectal cancer screening, 16.49 in men, 13.95 in women for gastric cancer screening, 16.54 in men, 13.95 in women for lung cancer screening. Logistic regression models were used to adjust multivariable ORs for fiscal years of baseline survey (2013, 2014, or 2015), educational level (low level, medium level, high level, or unknown), working status (regular employee, non‐regular employee or not working, or unknown), cohabitants (living with fewer than two people, living with three or more people, unknown), smoking status (never smokers, current smokers, past smokers, unknown), alcohol drinking (never drinkers, current drinkers, past drinkers, unknown), 24‐h urinary sodium excretion in grams/day (less than median, median or more, unknown), physical activity in metabolic equivalents (METs) hours/day (less than median, median or more, unknown), Body mass index in kg/m^2^ (< 18.5, 18.5–24.9, ≥ 25.0, unknown), comorbidities by self‐reported questionnaire (cancer, heart disease, stroke), depressive symptoms (none [Center for Epidemiological Studies Depression {CES‐D} score < 16], presence [CES‐D score ≥ 16], unknown). Item 2 of the Lubben Social Network Scale: How many relatives do you feel close to, such that you can talk about private matters? (fewer than two, three or more, or unknown).

Abbreviations: CI, confidence interval; OR, odds ratio.

## 4 Discussion

Using a community‐based biobank dataset from Japan, this study hypothesized that there would be high concordance in cancer screening attendance between spousal couples, regardless of cancer type, and that this concordance would not change based on the spouse's age.

The results of this study supported these hypotheses: (1) husbands' cancer screening attendance (as exposure) was significantly associated with their wives' attendance (as outcome); (2) similarly, wives' attendance (as exposure) was significantly associated with their husbands' attendance (as outcome); (3) these associations were consistent across all three types of cancer screenings; and (4) the associations remained significant when stratified by age (64 years or younger or 65 years or older) of the exposed spouse.

The concordance of colorectal cancer screening (colonoscopy) in 804 married couples was examined over 5 years [17], finding a significant association when one spouse attended screening (OR: 1.94, 95% CI: 1.39–2.67). Although the findings of the present study are consistent with this study, direct comparisons are limited as the previous study used colonoscopy, whereas colorectal cancer screening in Japan typically uses fecal occult blood testing [28]. Additionally, the previous study did not distinguish between the exposure and outcome spouse; however, both scenarios were analyzed in the present study, and high concordance was achieved.

Another Japanese study by Watanabe et al. investigated the concordance between husbands' health checkups (primary prevention) and wives' cervical or breast cancer screening (secondary prevention) in approximately 40,000 spousal couples [18]. They found that husbands' attendance at health checkups was significantly associated with wives' cancer screening attendance. The present study differs from the abovementioned study in that both exposure and outcome were cancer screening (secondary prevention), preventing a direct comparison.

The present study has three main strengths. First, spousal concordance was examined for three types of cancer screening (colorectal, gastric, and lung), demonstrating strong associations across all types. Previous studies have focused only on colorectal cancer screening. Second, analyses were performed with husbands as the exposure group and wives as the outcome group and vice versa. Strong concordance was found in cancer screening attendance regardless of which spouse was the exposure. Third, age‐associated variation in concordance was assessed, given that age is a known factor in cancer screening attendance, with higher attendance rates among older individuals [29, 30, 31]. The results provide new evidence that spousal concordance in cancer screening attendance does not differ by age group.

### 4.1 Study Limitations

The present study has some limitations. First, as a cross‐sectional study, the temporal relationship between exposure and outcomes was unclear. Second, cancer screening attendance was self‐reported, which can lead to misclassification owing to reliance on memory. Third, in Japan, cancer screening is categorized into “municipality‐based,” “workplace‐based,” and “voluntary” screenings. Future studies should include data on the type of screening entity to avoid limitations in conducting stratified analyses according to screening type. In an earlier study, spousal concordance remained consistent across different types of screening entities [18]. Fourth, marital status significantly influences cancer screening attendance [29, 30, 31]. Factors such as the duration of the marriage or the quality of the relationship could affect concordance. However, these variables could not be analyzed. Instead, the age of the exposed group was used as a proxy for marriage duration, and Item 2 of the Lubben Social Network Scale was used as a proxy for relationship quality [26, 27]. This approach means residual confounding could not be fully assessed owing to missing data. Furthermore, this study included couples who both participated in the study in the analysis. It could have been a better relationship between the couple. Therefore, our results may have been overestimations compared to those of couples who participated in the survey alone. Fifth, recruitment for this study was not as spouses but as individuals, and both spouses happened to participate (and were matched at that point); therefore, the selection bias in the concordance rate may be higher than that in the general population. Finally, in the type 2 survey of the Tohoku Medical Megabank, participants who voluntarily visited our assessment center were recruited. Meanwhile, in the Type 1 survey, Tohoku Medical Megabank investigators went to the local government's specific health check‐up venue to conduct the survey. Therefore, type 2 surveys might contain more voluntary participants than specific health check‐based surveys (type 1 surveys), and might not be representative of the general population that lived in Miyagi Prefecture. As we showed, the level of psychological distress (K6 ≥ 13) or current prevalence of smoking was lower than that of type 1 survey participants [20], suggesting that mental health or lifestyle might be better.

### 4.2 Clinical Implications

The results of this study are expected to encourage increased cancer screening attendance. A previous study in Japan demonstrated that behavioral interventions targeting individual participants significantly boosted breast cancer screening attendance [32]. Recently, interest has grown in interventions aimed at couples. Unlike individual‐based approaches, this study suggests that interventions targeting both spouses could create a ripple effect, leading to higher overall screening attendance [33]. Hence, if one or both spouses do not participate in cancer screening, couple‐based interventions may be more effective. Future research should explore strategies for implementing such interventions.

## 5 Conclusions

In this study, concordance between couples was analyzed for colorectal, gastric, and lung cancer screening attendance in a community‐based cross‐sectional study of spousal couples in Japan. Altogether, the findings support the hypothesis that the attendance of one spouse at cancer screening significantly positively influences the attendance of the other spouse, regardless of the type of cancer screening or the age of the spouse.

## Author Contributions

**Naoki Nakaya and Kumi Nakaya:** conceptualization. **Naoki Nakaya, Kumi Nakaya, Toshimasa Sone, and Atsushi Hozawa:** methodology. **Naoki Nakaya, Kumi Nakaya, and Toshimasa Sone:** formal analysis. **Naoki Nakaya, Kumi Nakaya, Mana Kogure, Rieko Hatanaka, Ippei Chiba, Sayuri Tokioka, Masato Takase, Nobuo Fuse, Yoko Izumi, and Atsushi Hozawa:** writing – original draft. **Atsushi Hozawa**: supervision.

## Ethics Statement

The study protocol adhered to the 1975 Declaration of Helsinki's ethical guidelines. The study protocol was reviewed and approved by the Ethics Committee of ToMMo, Tohoku University (Sendai, Japan) (initial approval: 2012‐4‐617; most recent revision: 2021‐4‐113). Written informed consent was obtained from participants.

## Conflicts of Interest

The authors declare no conflicts of interest.

## Supporting information

Supporting Information S1

## Data Availability

The Tohoku Medical Megabank (TMM) data‐sharing policy is publicly available at http://www.megabank.tohoku.ac.jp/english/sample/. A request to use TMM biobank data for research purposes should be made to the Tohoku Medical Megabank Organization (ToMMo) headquarters. All requests are subject to approval from the Sample and Data Access Committees. Details are available upon request from dist@megabank.tohoku.ac.jp.
