# Supplementary material for: Concordance of Cancer Screening Attendance Among Spouse Couples: A Cross‐Sectional Survey of the Tohoku Medical Megabank Project
Source: Psychooncology. 2025 Apr 25;34(5):e70158. doi: 10.1002/pon.70158 (PMC12031694; doi:10.1002/pon.70158)
Supplement: Supplementary file 1 — Supporting Information S1 [file PON-34-e70158-s001.docx]

**Supplements**

**Doc. S1**

**Contents of colon, stomach, and lung cancer screenings used in this study**

The Ministry of Health, Labour, and Welfare, Japan, has established guidelines for the above three cancer screenings and promotes cancer screening by municipalities based on scientific evidence.

1. Colorectal cancer screening

- Screening content is an interview and fecal occult blood test

- Target participants are 40 years old or older

- Screening is once a year

2. Gastric cancer screening

- Screening content is an interview and either stomach X-ray or gastroscopy

- Target participants are 50 years or older (stomach X-rays can be performed on those 40 years or older)

- Screening is twice a year (stomach X-ray can be performed once a year)

3. Lung cancer screening

- Screening content is an interview, chest X-ray, and sputum cytology

- Target participants are 40 years or older (sputum cytology is generally for heavy smokers aged 50 years or older)

- Screening is once a year

**Estimated 24-hour urinary sodium excretion**

The estimated 24-hour urinary sodium excretion (24HUNaV) was calculated using the Tanaka formula as follows:

24HUNaV [mEq/day] = 21.98 × XNa0.392

PRCr [mg/day] = −2.04 × age + 14.89 × weight [kg] + 16.14 × height [cm] − 2244.45

where XNa [mEq/day] = (SUNa [mEq/L] /SUCr [mg/dl] /10) × PRCr [mg/day]

PRCr = predicted value of 24-hour urinary creatinine excretion

SUNa = sodium concentration in the spot urine

SUCr = creatinine concentration in the spot urine

**Physical activity (PA) in MET hours/day**

PA levels were assessed using three questionnaires: “daily PA,” “leisure-time PA,” and “sleeping duration.” The total MET hours/day was the sum of MET hours/day from those three questionnaires.

In the first “daily PA” questionnaire, participants were asked about the average daily time spent doing heavy physical work, walking, standing, or sedentary. The representative time hour values for none, <0.5 hour, 0.5 ≤ 1 hour, 1 ≤ 3 hours, 3 ≤ 5 hours, 5 ≤ 7 hours, 7 ≤ 9 hours, 9–11 hours, 11+ hours were 0, 0.5, 0.5, 2, 4, 6, 8, 10, and 11 hours, respectively. MET hours/day were estimated by multiplying the daily time score for each activity by the MET intensity of that activity: 4.5 for heavy physical work or strenuous exercise; 2.0 for standing or walking; and 1.5 for being sedentary.

In the second questionnaire on “leisure-time PA.” MET hours/day were determined using a self-administered questionnaire. Participants were asked about the frequency and average duration of leisure-time PAs according to four broad intensity categories (vigorous, moderate, light, or slight). Vigorous activities, defined as those that cause a person to breathe more heavily than normal (to the extent that they cannot talk), were allocated 4.5 METs; moderate activities, defined as those that cause a person to breathe somewhat more heavily than normal (to the extent that they can still talk), were allocated 4.0 METs; light activities, defined as those that cause a person to breathe normally (such as walking), were allocated 4.0 METs; and slight activities, defined as those that cause person to breathe normally (such as slow walk), were allocated 3.0 METs. The frequency categories (assigned average days per week) for leisure-time PA were: almost none (0), one to three times per month (0.1), one to two times per day (0.2), three to four times per day (0.5), and every day (1.0). The average duration categories (assigned average hours per activity) were: <30 min (0.3), 30 min ≤ 1 hour (0.75), 1 ≤ 2 hours (1.5), 2 ≤ 3 hours (2.5), 3 ≤ 4 hours (3.5), and ≥4 hours (4.0). A MET-hour per week of leisure-time PA for each category of intensity was calculated using the daily frequency, duration, and intensity of leisure-time PA, according to the formula:

Leisure time PA (MET·h·wk−1) = intensity of PA (METs) × duration (h) × frequency (d·wk−1)

In the third questionnaire on “sleeping duration,” participants were asked about the average sleeping duration per day: <5 hours (2.5), 5 ≤ 6 hours (5.5), 6 ≤ 7 hours (6.5), 7 ≤ 8 hours (7.5), 8 ≤ 9 hours (8.5) and ≥9 hours (10.0). MET hours/day were estimated by multiplying the daily sleeping duration by the MET intensity of sleeping, which was 0.9.
